# Supplementary material for: C-type natriuretic peptide attenuates enhanced glycolysis and de novo pyrimidine synthesis in pericytes of patients with pulmonary arterial hypertension
Source: Commun Biol. 2025 Aug 12;8:1199. doi: 10.1038/s42003-025-08661-0 (PMC12343844; doi:10.1038/s42003-025-08661-0)

Noh et al.

Supplementary Material

Supplementary Figures:
Supplementary Figure 1:

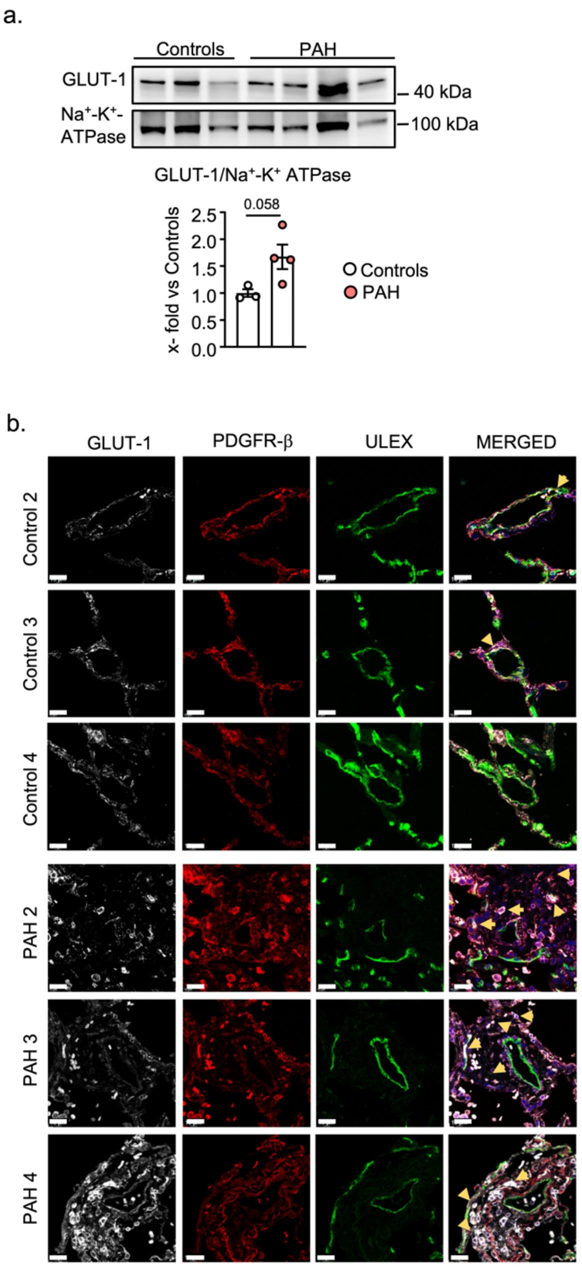

Supplementary Figure 1: Membrane GLUT-1 expression is higher in PAH pericytes compared to controls. (a) In comparison to control lung pericytes, enriched membrane fractions from PAH pericytes exhibit higher GLUT-1 protein (p value - 0.058). (n =3 controls and n = 4 PAH patients; unpaired 2-tailed Student's t test). **(b)** Immunofluorescence staining on control and IPAH patient lung tissues (n = 3 from each) using GLUT-1 (white), PDGFR-β (red: pericyte marker), ULEX (green: endothelial stain), and nuclei were stained by DAPI (blue). Arrows indicate the colocalization of PDGFR-β with GLUT-1. Scale bar = 20 μm. Data is presented as Mean ± SEM.

Supplementary Figure 2:

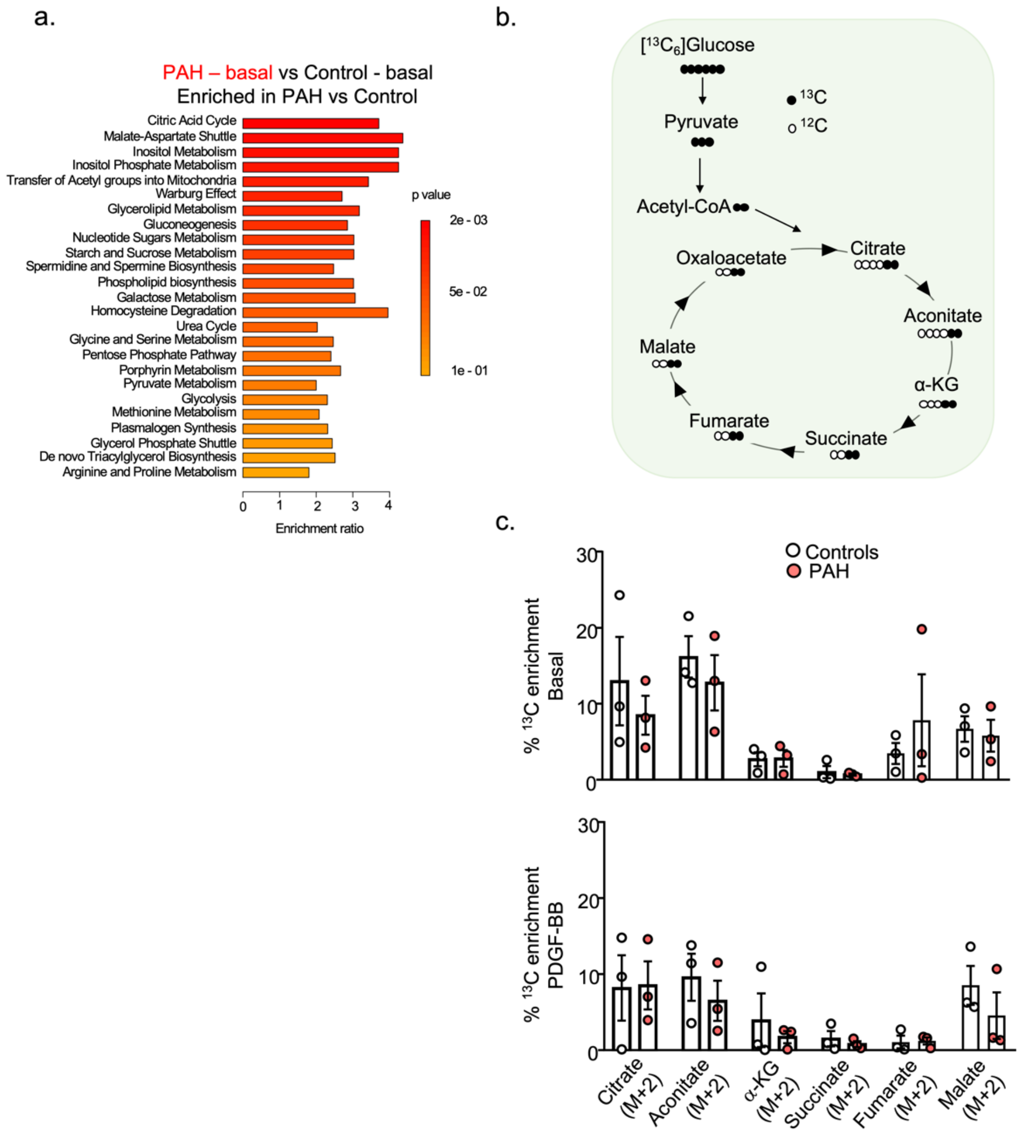

Supplementary Figure 2: TCA cycle flux is unchanged between control and PAH pericytes. (a) Metabolic pathways enriched in PAH pericytes versus controls at baseline based on metabolite set enrichment analysis using MetaboAnalyst 6.0. The corresponding fold enrichments and computed p values are depicted. **(b)** Schematic representation of ^{13}C labelling patterns after the metabolism of $^{13}C_6$ -glucose through Tricarboxylic acid (TCA) cycle **(c)** Percentage ^{13}C enrichment in the TCA cycle intermediates indicate no change in isotope enrichment between control and PAH pericytes under basal conditions and PDGF-BB stimulation. Data are shown as percent isotope enrichment, normalized to the total signal (M+0 to M+n). (n = 3 controls and PAH pericytes, unpaired student's t - test). Data is presented as Mean ± SEM.

Supplementary Figure 3:

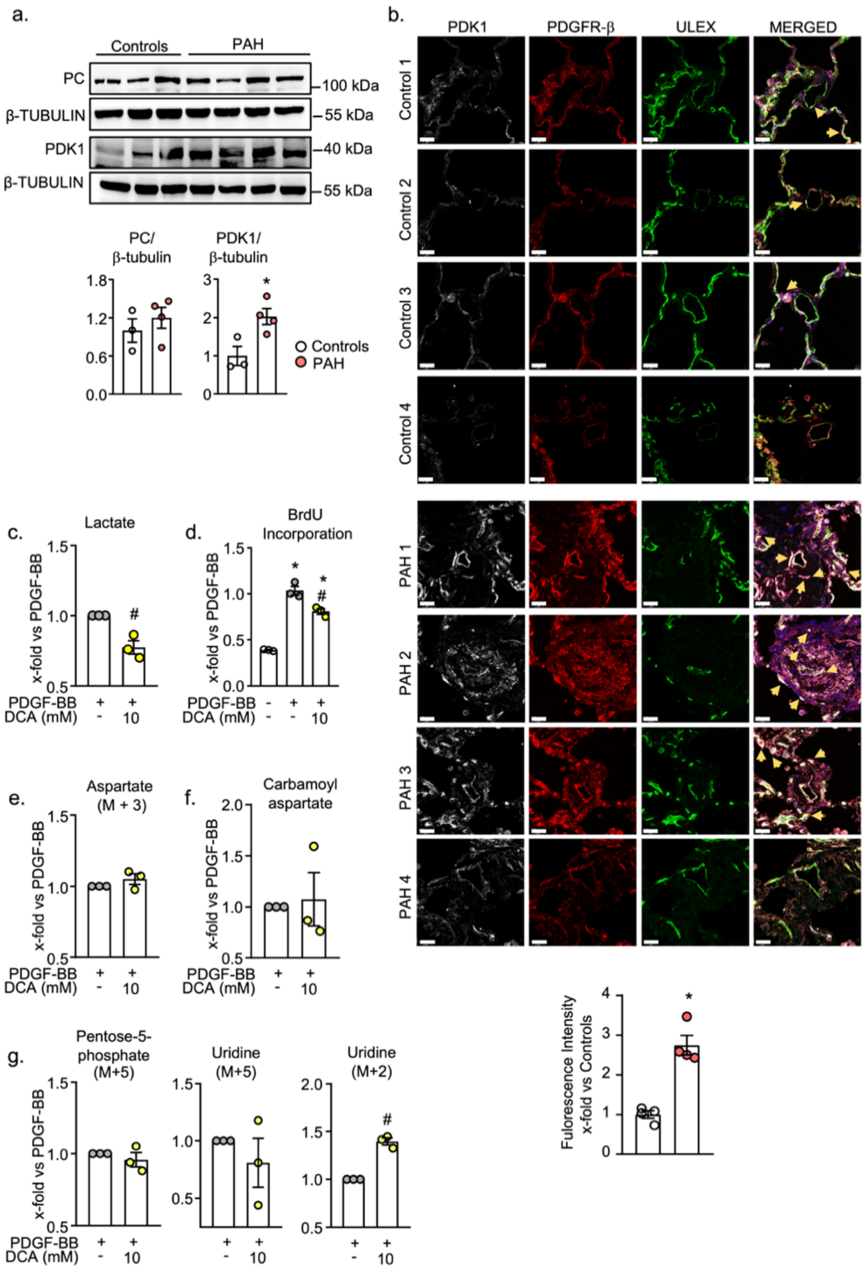

Supplementary Figure 3: PDK1 expression is strongly upregulated in PAH pericytes. (a) PDK1 expression is significantly upregulated in cultured PAH pericytes compared to controls while PC expression remains unchanged (n =3 controls and n = 4 PAH patients; unpaired 2-tailed Student's t test). **(b)** Immunofluorescence staining of PDK1 on control (n = 4) and IPAH patient lung tissues (n = 4), followed by fluorescence quantification. PDK1 (white), PDGFR-β (red: pericyte marker), ULEX (green: endothelial stain), and nuclei were stained by DAPI (blue). Arrows indicate the colocalization of PDGFR-β with PDK1. Scale bar = 20 µm. **(c - g)** DCA pretreatment (10 mM, 30min) significantly reduced **(c)** lactate levels and **(d)** PDGF-BB indcued proliferation in PAH pericytes but did not decrease **(e)** aspartate M + 3 labeling, **(f)** carbamoyl aspartate levels and **(g)** M + 5 and M +2 pyrimidine nucleotide labeling. n = 3 PAH pericytes, c, e, f and g: one sample t - test, d. 1-way ANOVA. For **a and b**: *p<0.05 vs Controls. For **c - g**: #p<0.05 vs PDGF-BB. For **d**: *p<0.05 vs PBS, #p<0.05 vs PDGF-BB. Data is presented as Mean ± SEM.

Supplementary Figure 4:

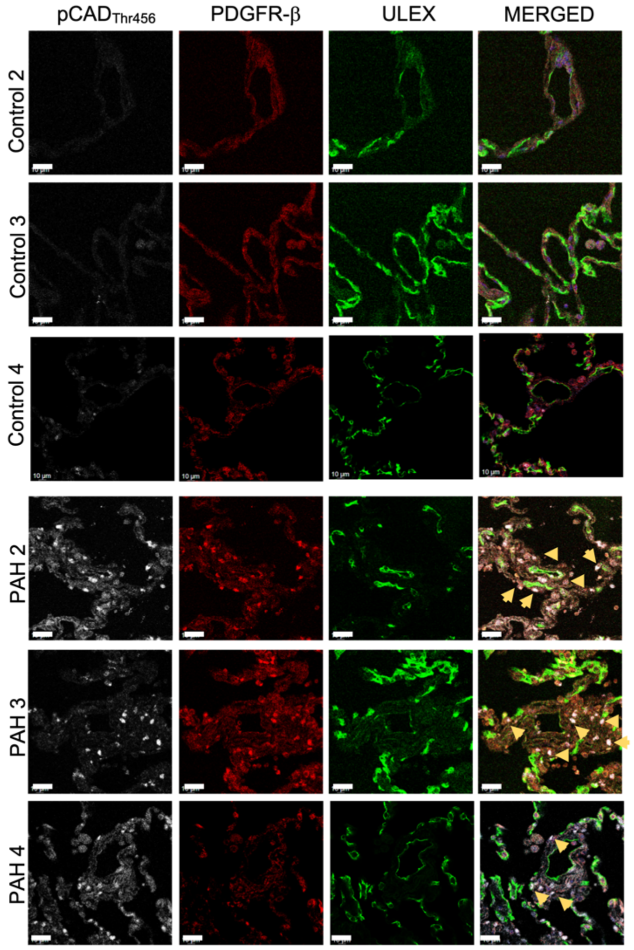

Supplementary Figure 4: Immunofluorescence staining on control (n = 3) and IPAH patient lung tissues (n = 3) using pCAD$_{Thr456}$ (white), PDGFR-β (red: pericyte marker), ULEX (green: endothelial stain), and nuclei were stained by DAPI (blue). Arrows indicate the colocalization of PDGFR-β with pCAD$_{Thr456}$. Scale bar = 20 µm.

Supplementary Figure 5:

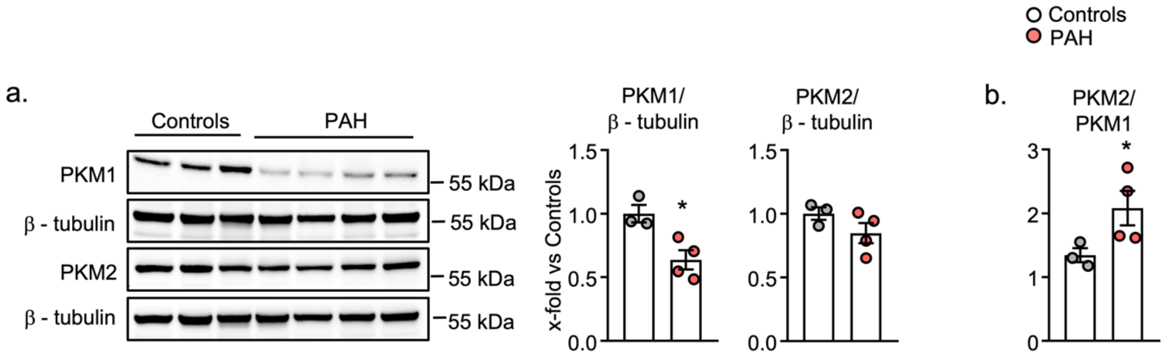

Supplementary Figure 5: PKM1 expression is downregulated in PAH pericytes, increasing PKM2/PKM1 ratio. (a - b) PKM1 expression was significantly downregulated in cultured PAH pericytes compared to controls with no difference in PKM2 expression resulting in an increased PKM2/PKM1 ratio. (n =3 controls and n = 4 PAH patients; unpaired 2-tailed Student’s t test). *p<0.05 vs Controls. Data is presented as Mean ± SEM.

Supplementary Figure 6:

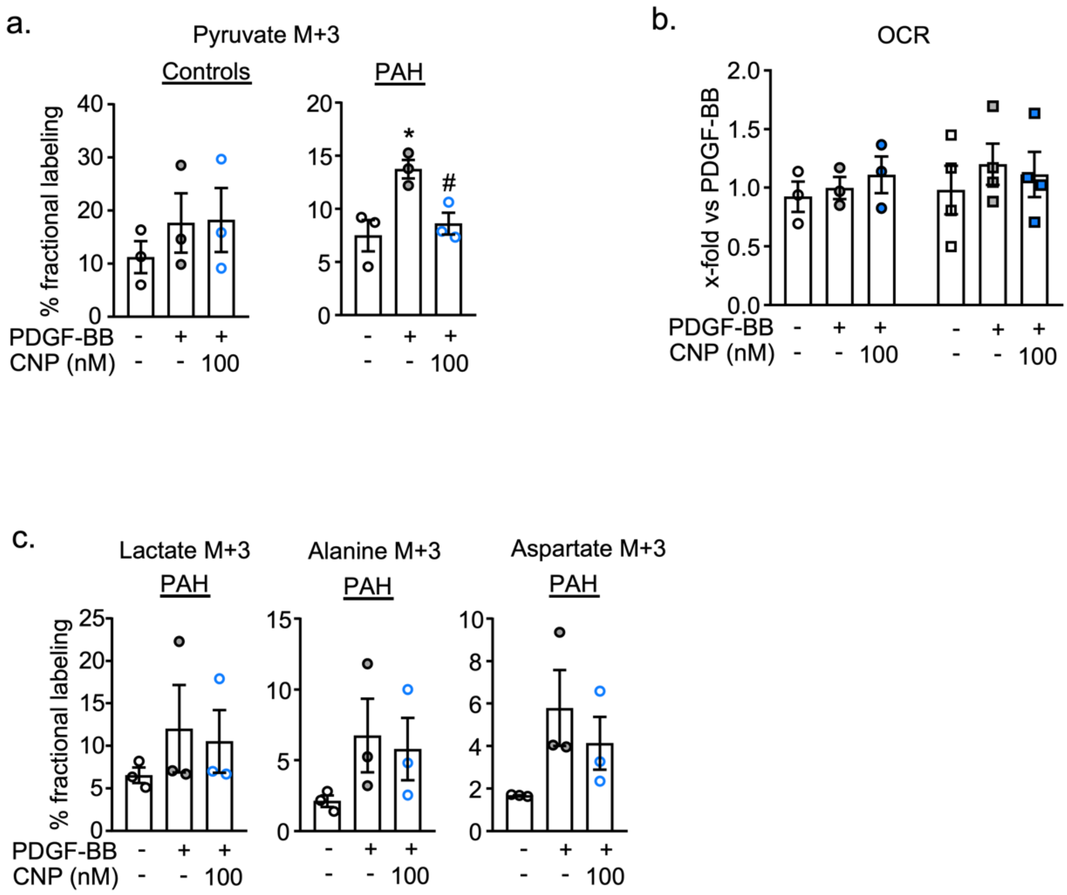

Supplementary Figure 6: CNP attenuates PDGF-BB induced Pyruvate M + 3 labeling in PAH pericytes. **(a)** CNP attenuates PDGF-BB induced increase in Pyruvate M + 3 labeling in PAH pericytes but not in controls (n = 3, 1-way ANOVA) **(b)** Oxygen consumption rate is unaltered by PDGF-BB +/- CNP in control and PAH pericytes. (n = 3 controls and n = 4 PAH pericytes, 1-way ANOVA) **(c)** mole fractions of M + 3 lactate, alanine and aspartate in PAH pericytes (n = 3, 1-way ANOVA). *$p < 0.05$ vs. PBS (–), #$p < 0.05$ vs. PDGF-BB. Data is presented as Mean ± SEM.

Supplementary Figure 7:

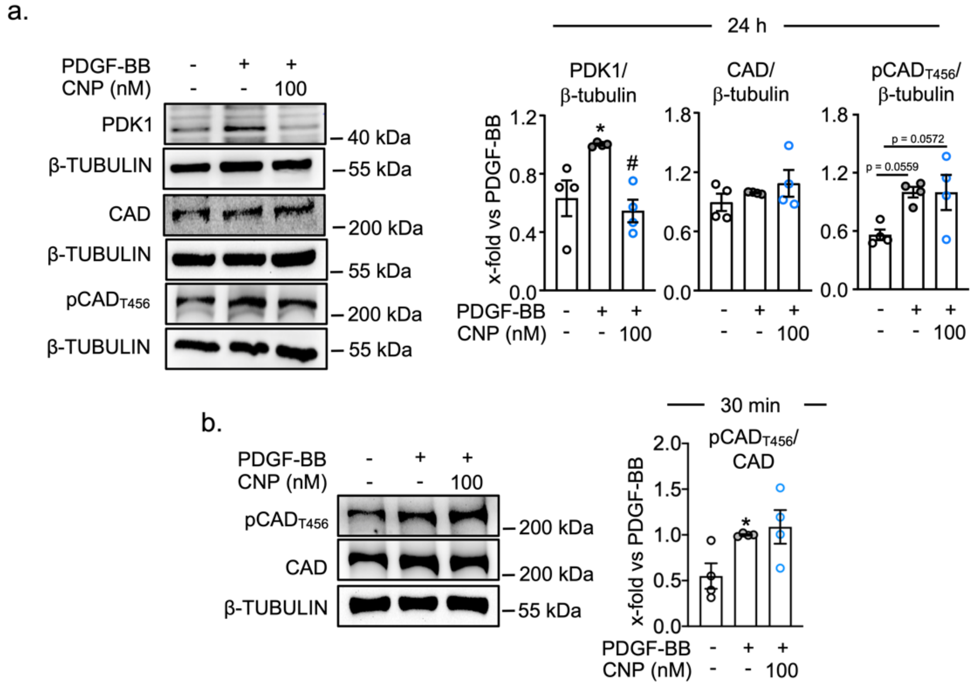

Supplementary Figure 7: CNP attenuates PDGF-BB induced PDK1 expression but not CAD in control pericytes. **(a)** PDGF-BB (30 ng/ml, 24 h) stimulation increases PDK1 and CAD phosphorylation at Threonine 456 with no effect on CAD expression. CNP reduced PDK1 expression (n = 4 control pericytes; 1-way ANOVA). **(b)** CNP does not prevent PDGF-BB (30 ng/ml, 30 min) induced CAD phosphorylation at Threonine 456 (n = 4 control pericytes, 1-way ANOVA). *p < 0.05 vs. PBS (–), #p < 0.05 vs. PDGF-BB. Data is presented as Mean ± SEM.

Supplementary Figure 8:

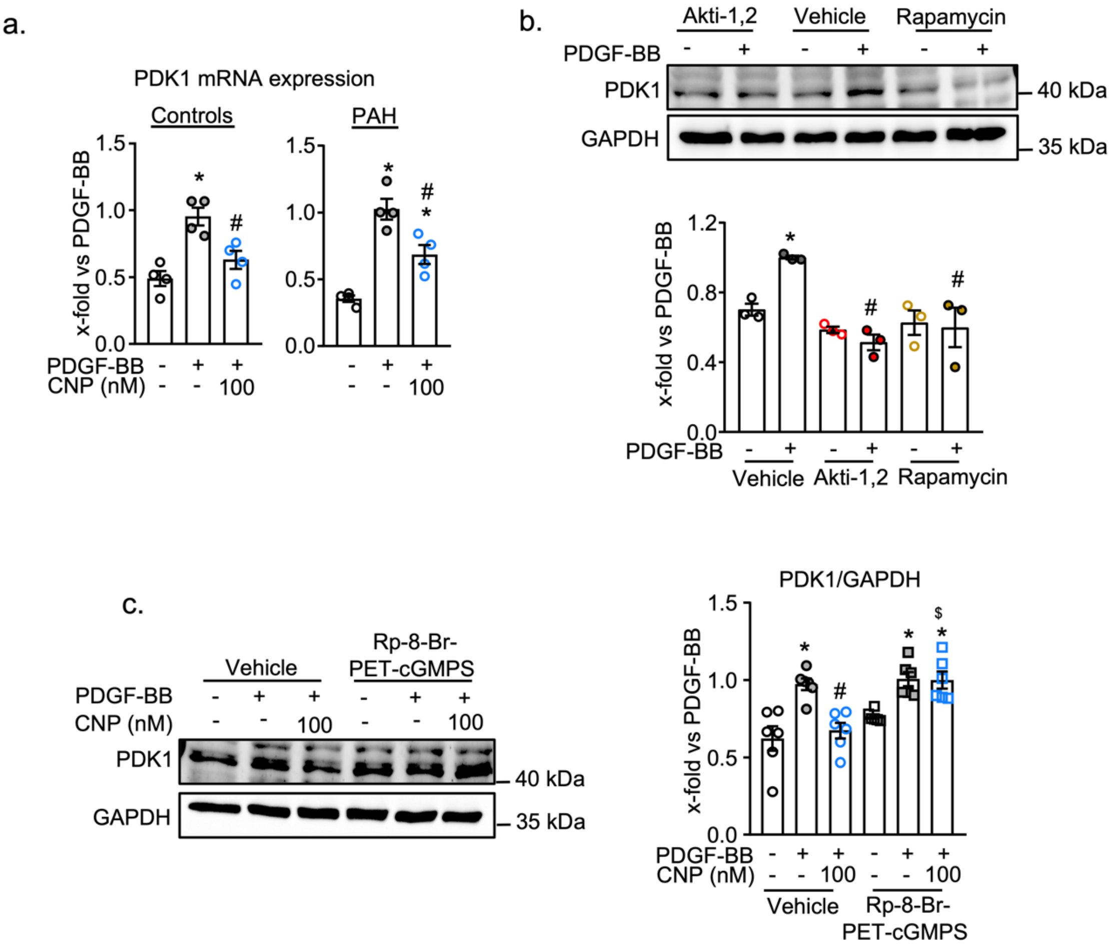

Supplementary Figure 8: CNP prevents PDGF-BB induced PDK1 expression in a cGKI- HIF-1α dependent manner. (a) CNP (100 nM, 30 min pretreatment) prevents PDGF-BB (30 ng/ml, 24 h) induced PDK1 mRNA expression in control (**a,** left panel) and PAH pericytes (**a,** right panel). (n = 4 controls and PAH, 1-way ANOVA). **(b)** AKT inhibitor (Akti-1, 5 µM) and mTORC inhibitor (Rapamycin, 100nM) prevent PDGF-BB induced PDK1 expression in control pericytes (n = 3; 1-way ANOVA). **(c)** cGKI inhibitor Rp-8-Br-PET-cGMPS (10 µM, 20 min pretreatment), prevented the effect of CNP on PDGF-BB (30 ng/ml, 24 h) - induced PDK1 expression (n = 6 biological replicates; 2-way ANOVA). For **a and b**: *p<0.05 vs PBS, #p<0.05 vs PDGF-BB. For **c:** *p<0.05 vs PBS (-), #p<0.05 vs PDGF-BB, \$p<0.05 vs corresponding vehicle-treated group (-). Data is presented as Mean ± SEM.

Supplementary tables:

Table 1: List of inhibitors

**Name**	**Company**	**Concentration**
GLUT-1 inhibitor (BAY-876)	Sigma (SML1774)	100 nM
cGKI inhibitor (Rp-8-Br-PET-cGMPS)	Biolog (P007)	1 μM
PDE2 inhibitor (BAY60-7550)	Gift from Prof. Johannes-Peter Stasch	100 nM
mTORC inhibitor (Rapamycin)	Selleck chemical (S1039)	100 nM
AKT inhibitor (AKTi-1/2)	Selleck chemicals (S7776)	5 μM
EPAC inhibitor (ESI-09)	Selleck chemicals (S7499)	1 μM
MEK inhibitor (PD98059)	Selleck chemicals (S1177)	10 μM

Table 2: List of antibodies

**Name**	**Company**	**Concentration**
GLUT-1	Cell Signaling	1: 1000 (12939S)
Na+-K^+ ATPase	Abcam	1: 1000 (ab26020)
PDK1	Proteintech	1: 1000 (18262-1-AP)
PKM1	Proteintech	1:1000 (15821-1-AP)
PKM2	Proteintech	1:1000 (15822-1-AP)
CAD	Santa Cruz	1: 1000 (sc-376072)
Phospho CAD (Thr456)	Santa Cruz	1: 1000 (sc-377559)
β – Tubulin	Proteintech	1: 1000 (10094-1-AP)
Phospho ERK 1/2 (Thr202/Tyr204)	Cell Signaling	1: 1000 (9102)
ERK	Cell Signaling	1: 2000
GAPDH	Cell Signaling	1:10000 (2118S)
NG2	Sigma (ZRB5320)	1:50 Immunofluorescence
PCNA	Santa Cruz	1:50 (sc:56) Immunofluorescence 1:1000 (sc-56) Immunoblotting
PDE2A	FabGennix	1: 1000 (PD2A-101A)
HIF-1α	Abcam	1: 1000 (ab179483)

Table 3: List of siRNA

**Name**	**Company**	**Sequence**
CAD siRNA	ThermoScientific (VHS41092)	Sense:GCUGGUCCAGAAUGGAACAtt Antisense: UGUUCCAUUCUGGACCAGCtt
Control siRNA	Qiagen (10277281)	Proprietary sequence

Table 4: List of primers

**Gene name**	**Forward primer**	**Reverse primer**
B2M	CACCCCCACTGAAAAAGATGAG	CCTCCATGATGCTGCTTACATG
SLC2A1 (gene encoding GLUT-1)	CTGTCGTGTCGCTGTTTGTG	AAAGATGGCCACGATGCTCA

PDK1	CTCAGGACACCATCCGTTCA	ATCTTGCAGGCCATACAGCA

Figure 1: Uncropped western blots

Figure 1f

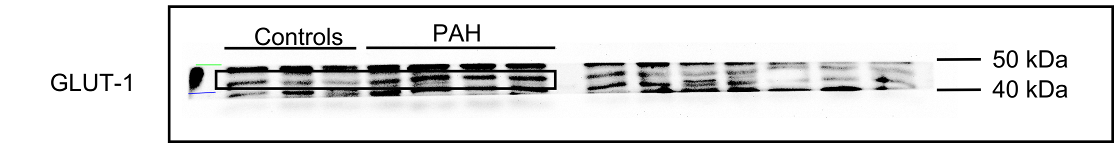

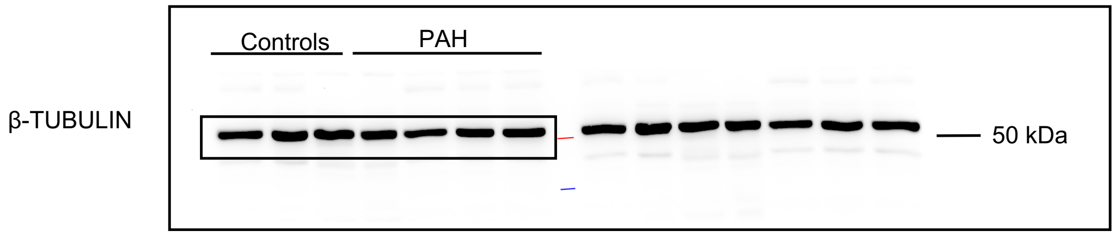

Figure 2: Uncropped western blots

Figure 2f

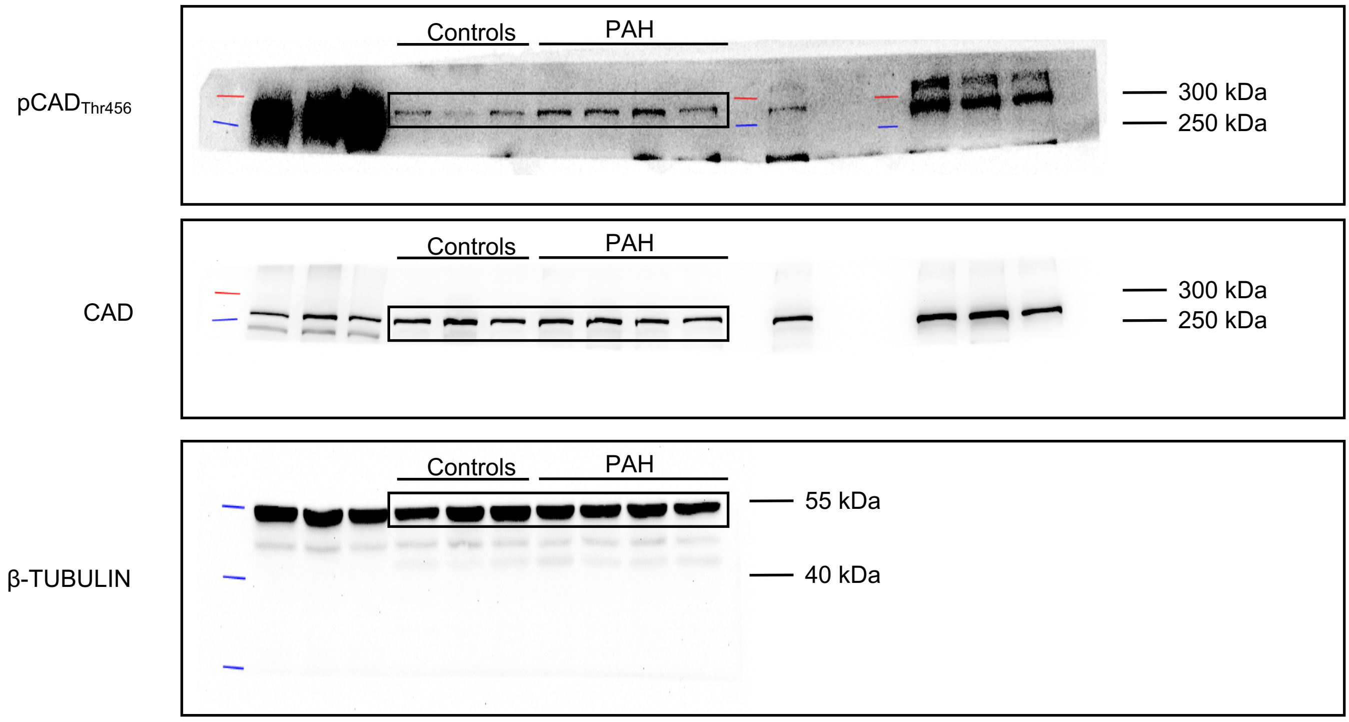

Figure 2h

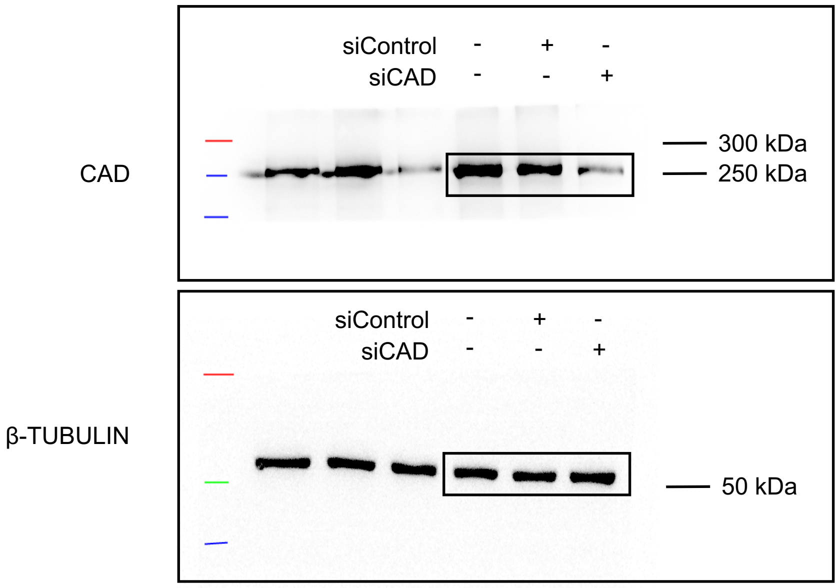

Figure 3: Uncropped western blots

Figure 3d

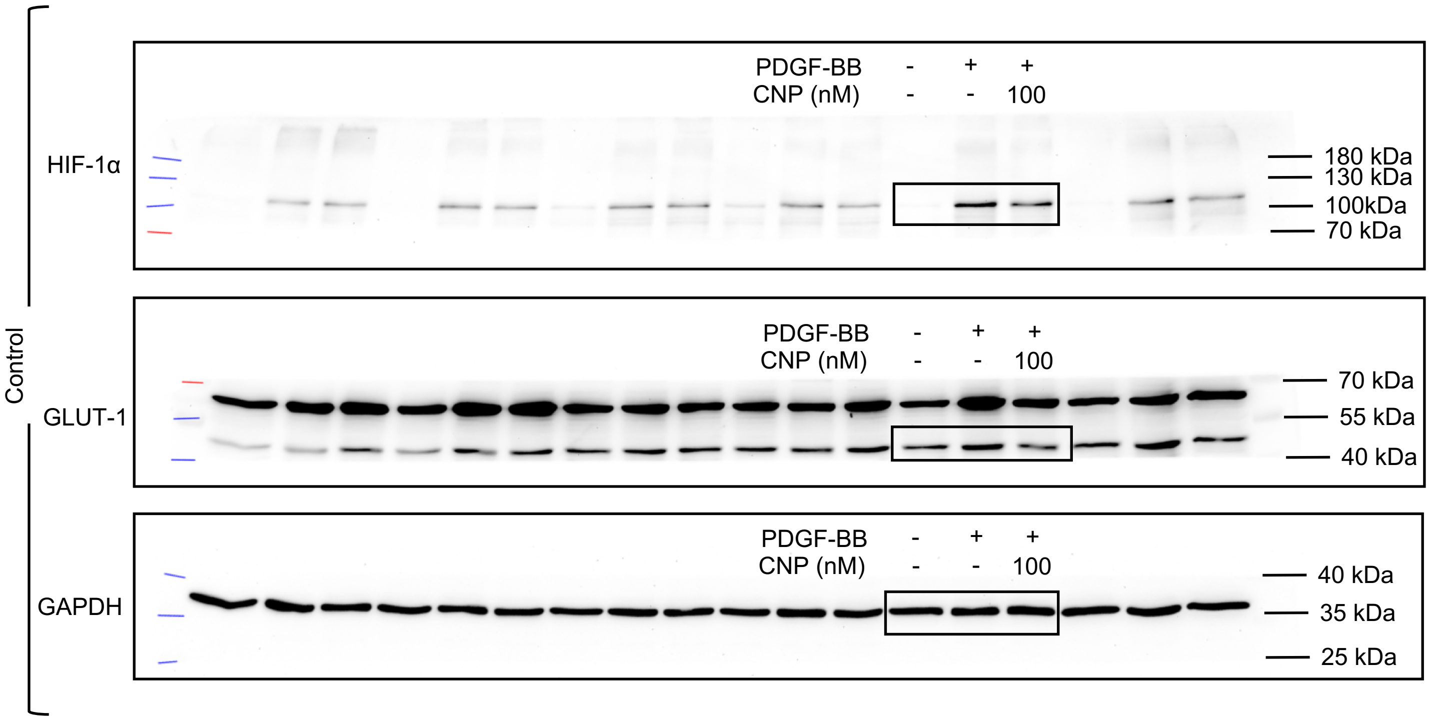

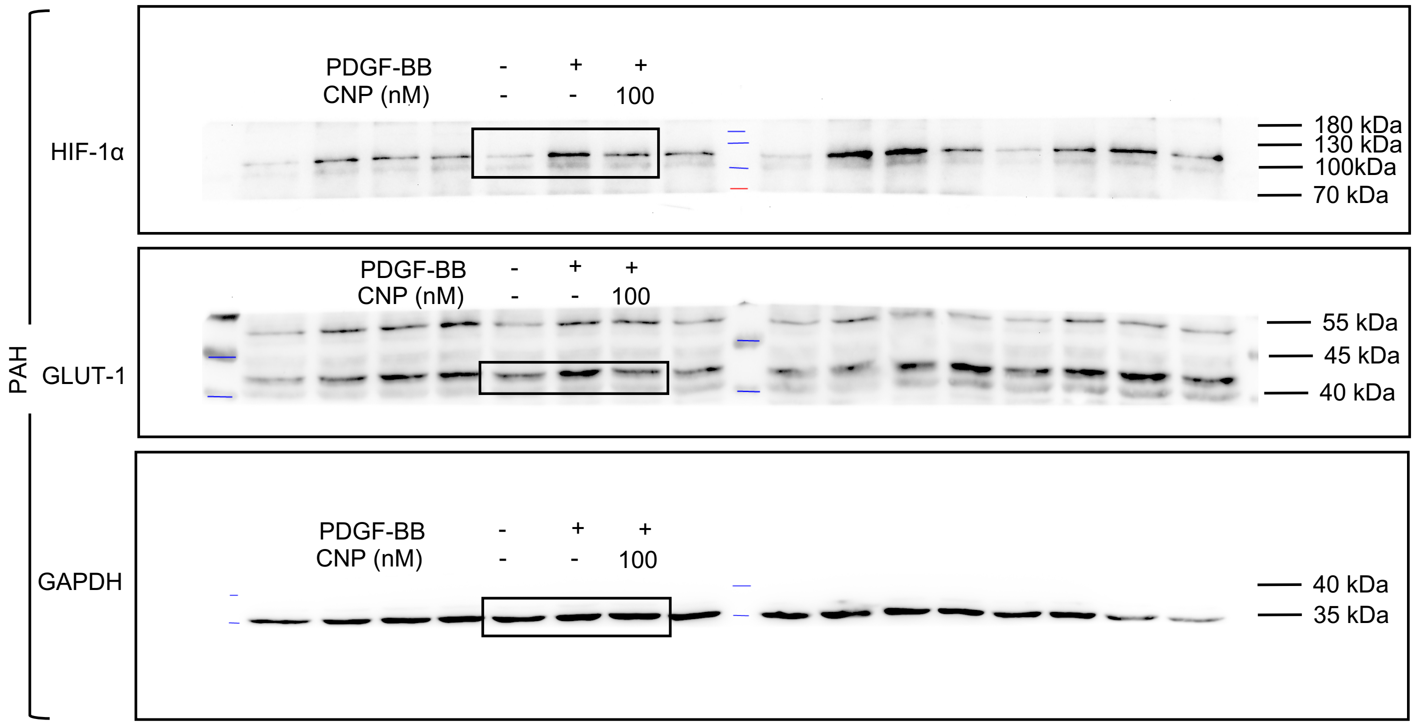

Figure 3: Uncropped western blots

Figure 3f

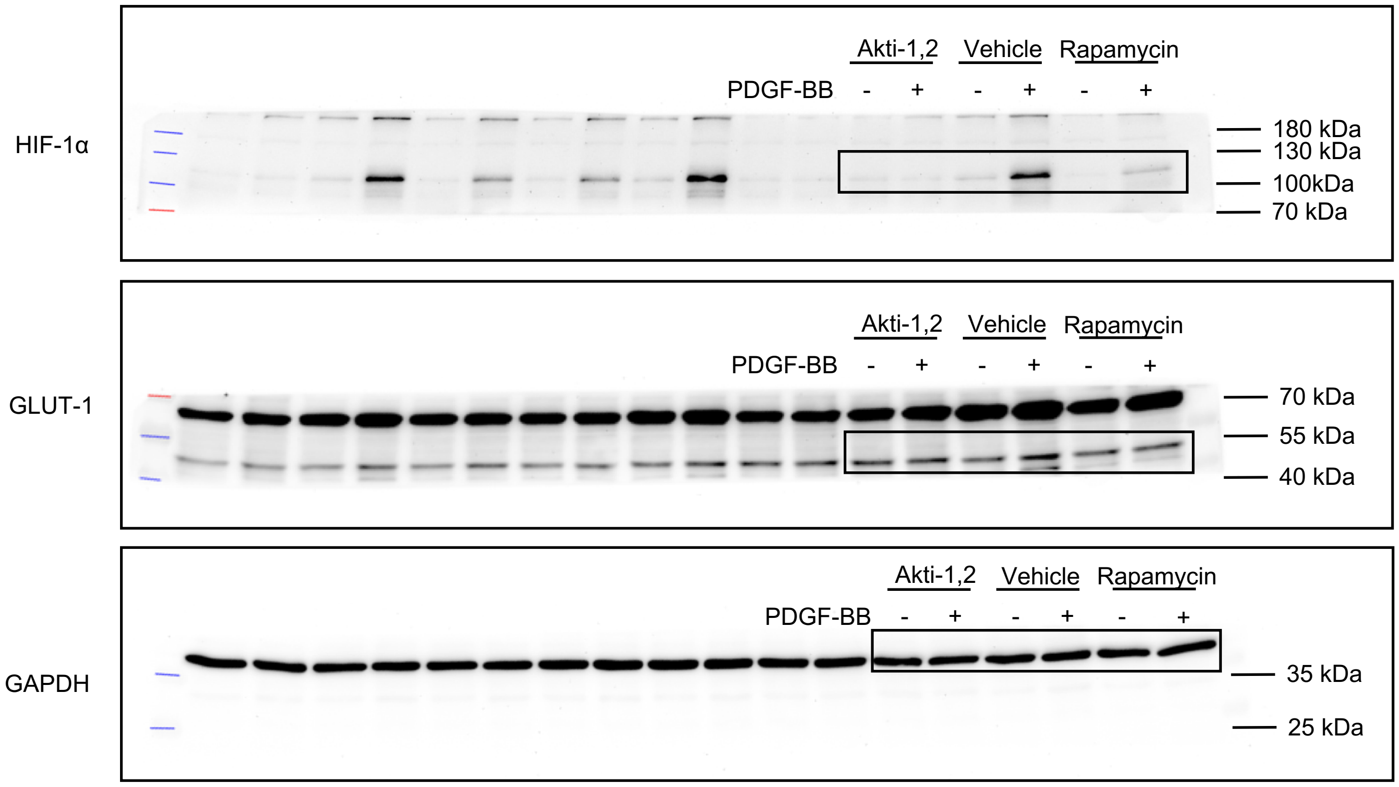

Figure 3g

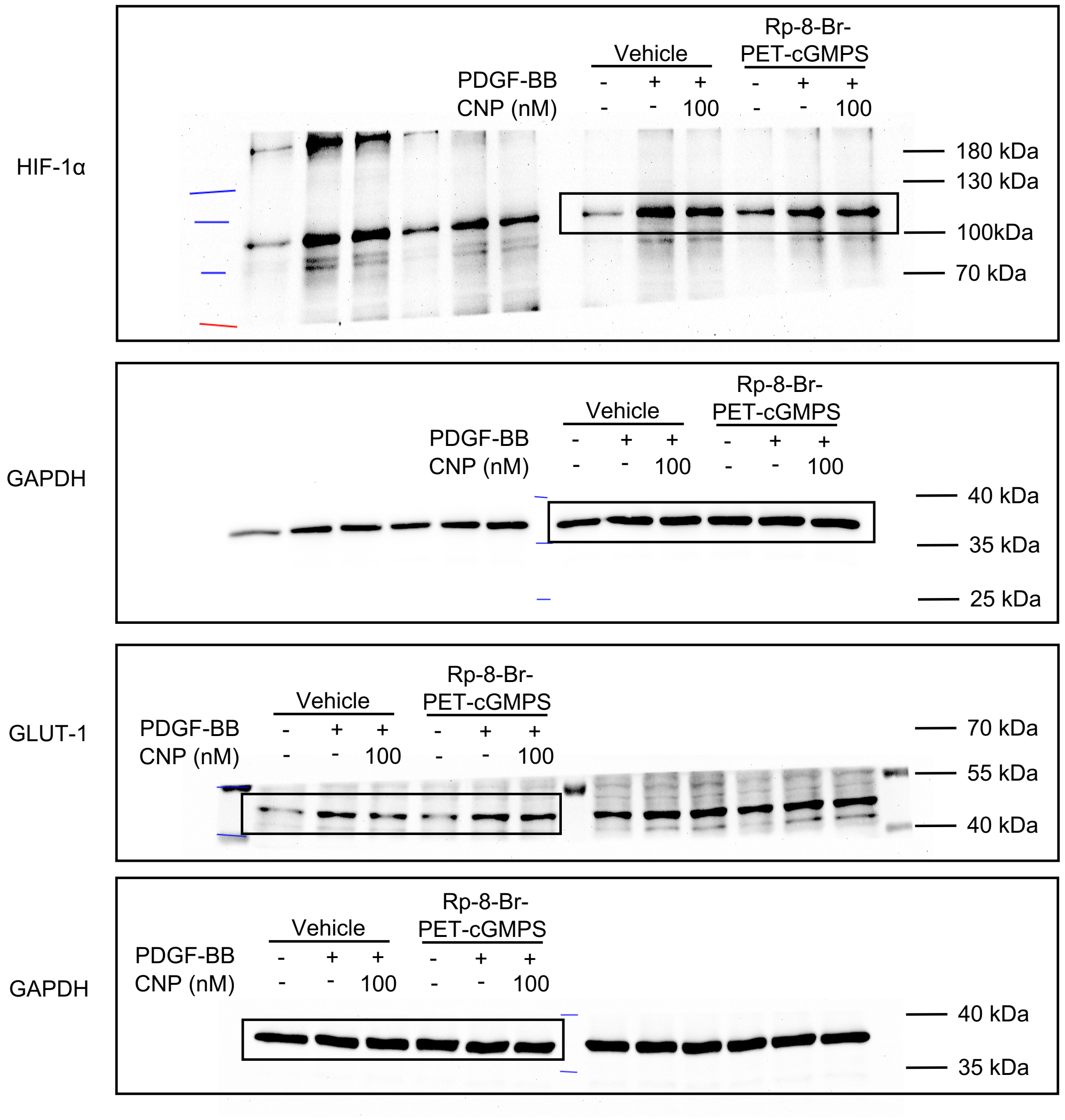

Figure 4: Uncropped western blots

Figure 4i

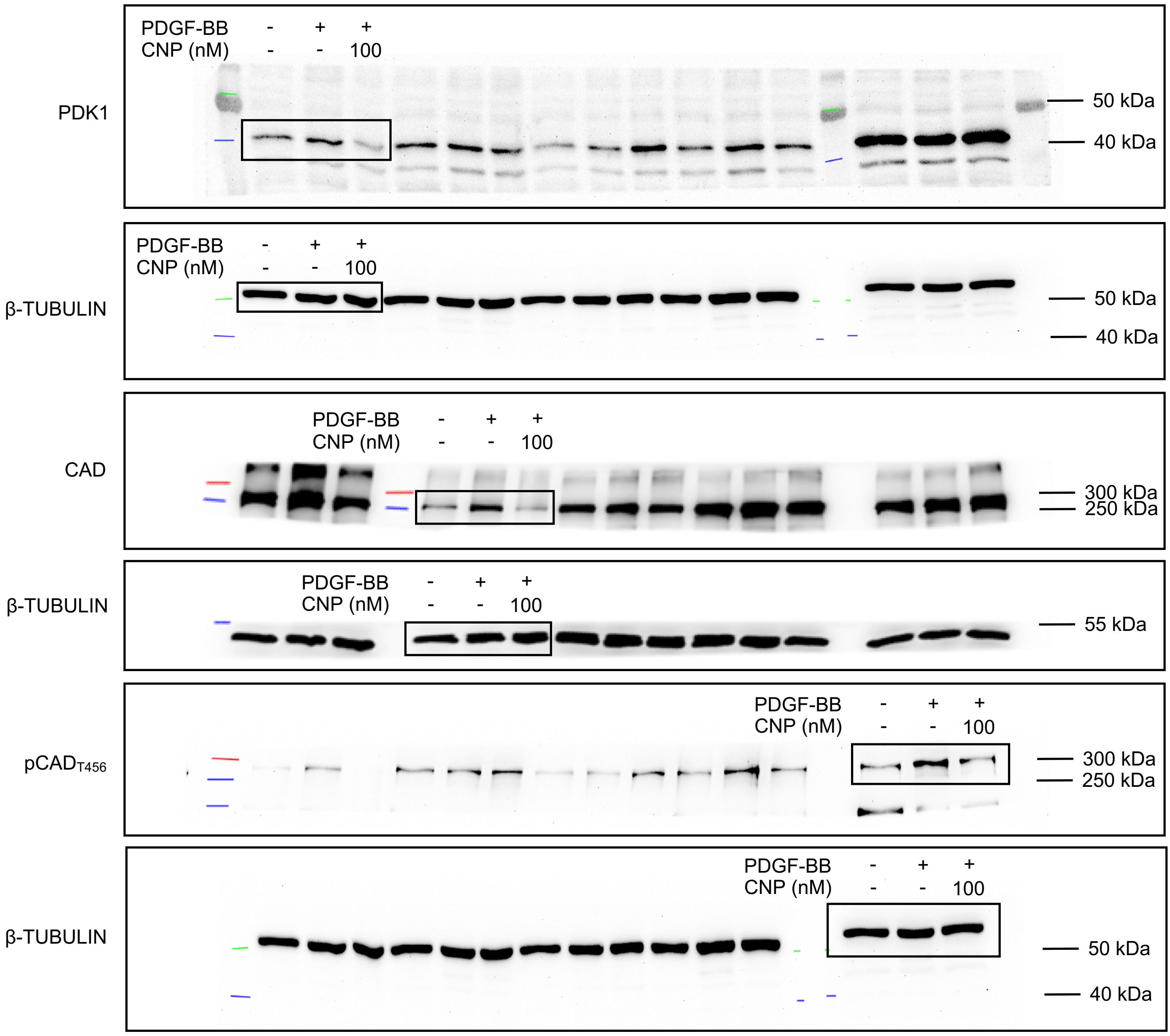

Figure 4j

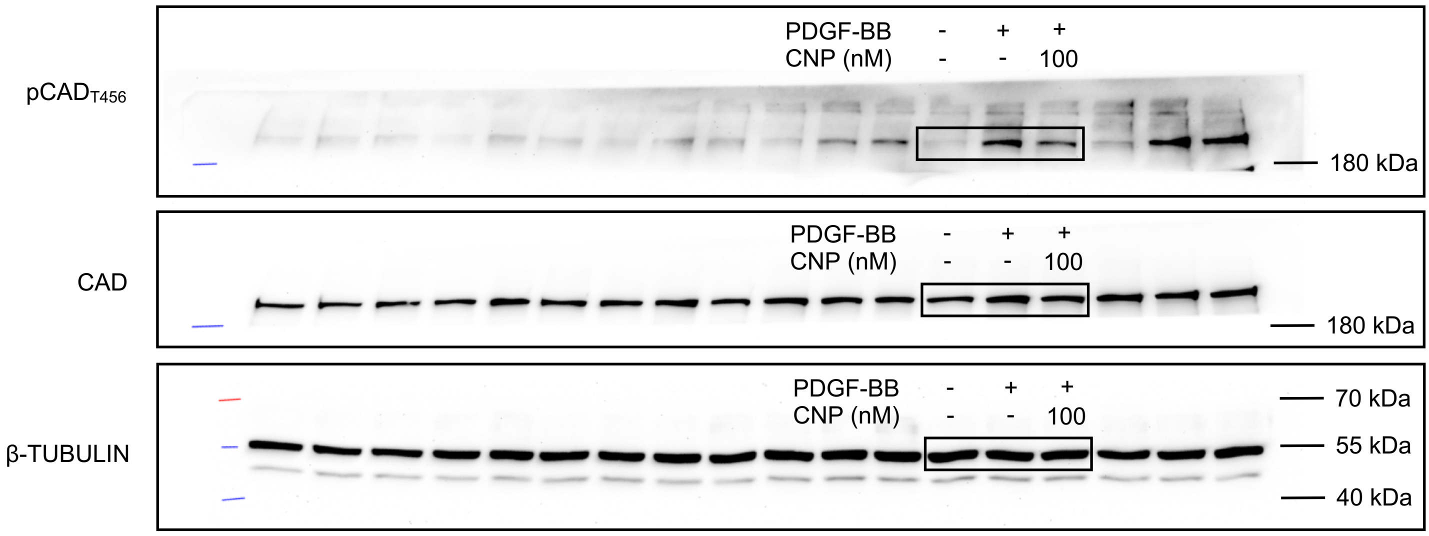

Figure 5: Uncropped western blots

Figure 5a

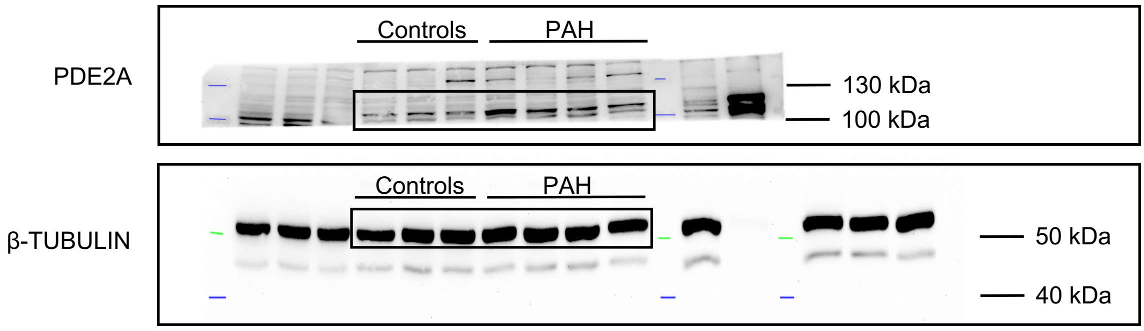

Figure 5f

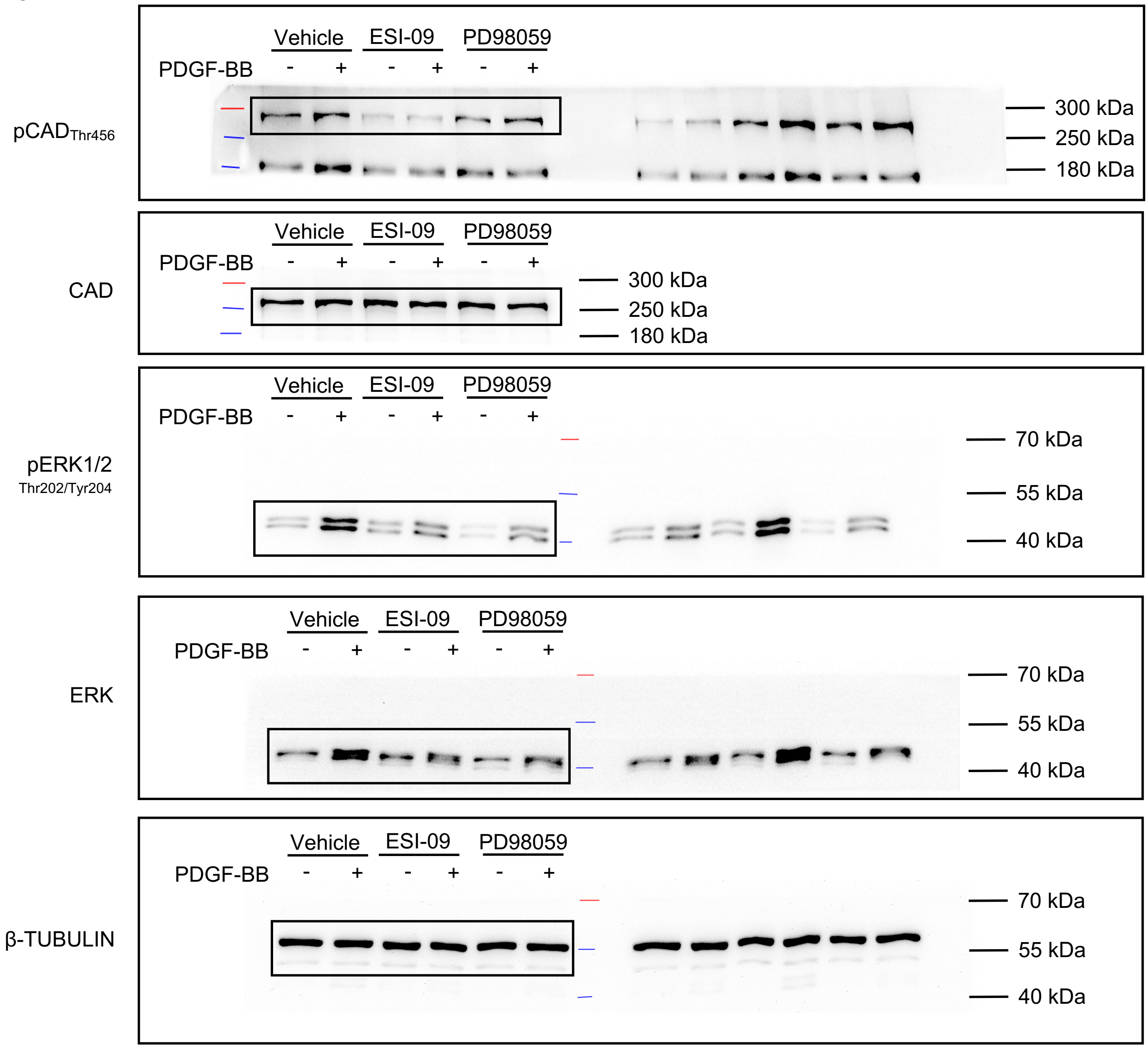

Figure 5: Uncropped western blots

Figure 5g

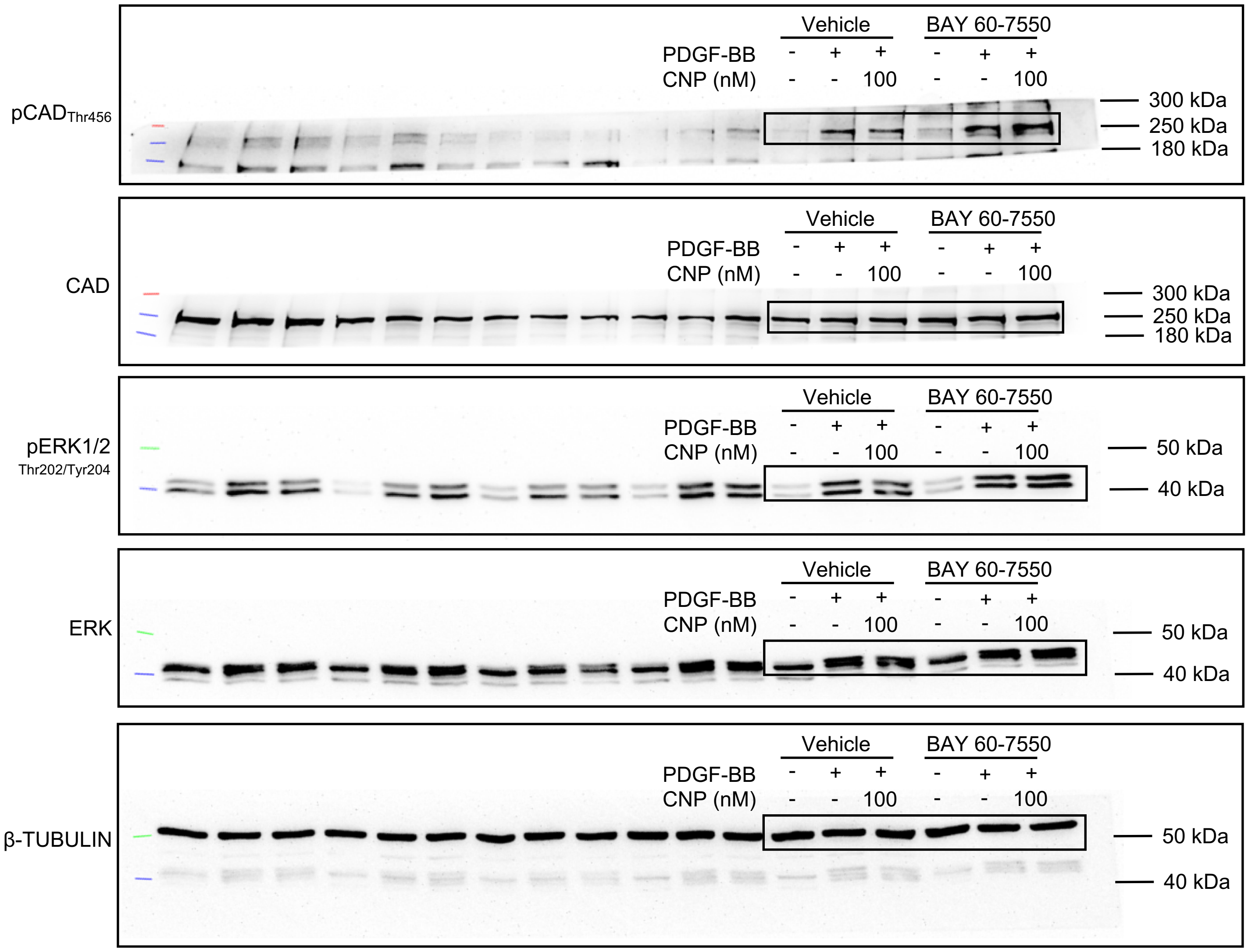

Figure 6: Uncropped western blots

Figure 6d

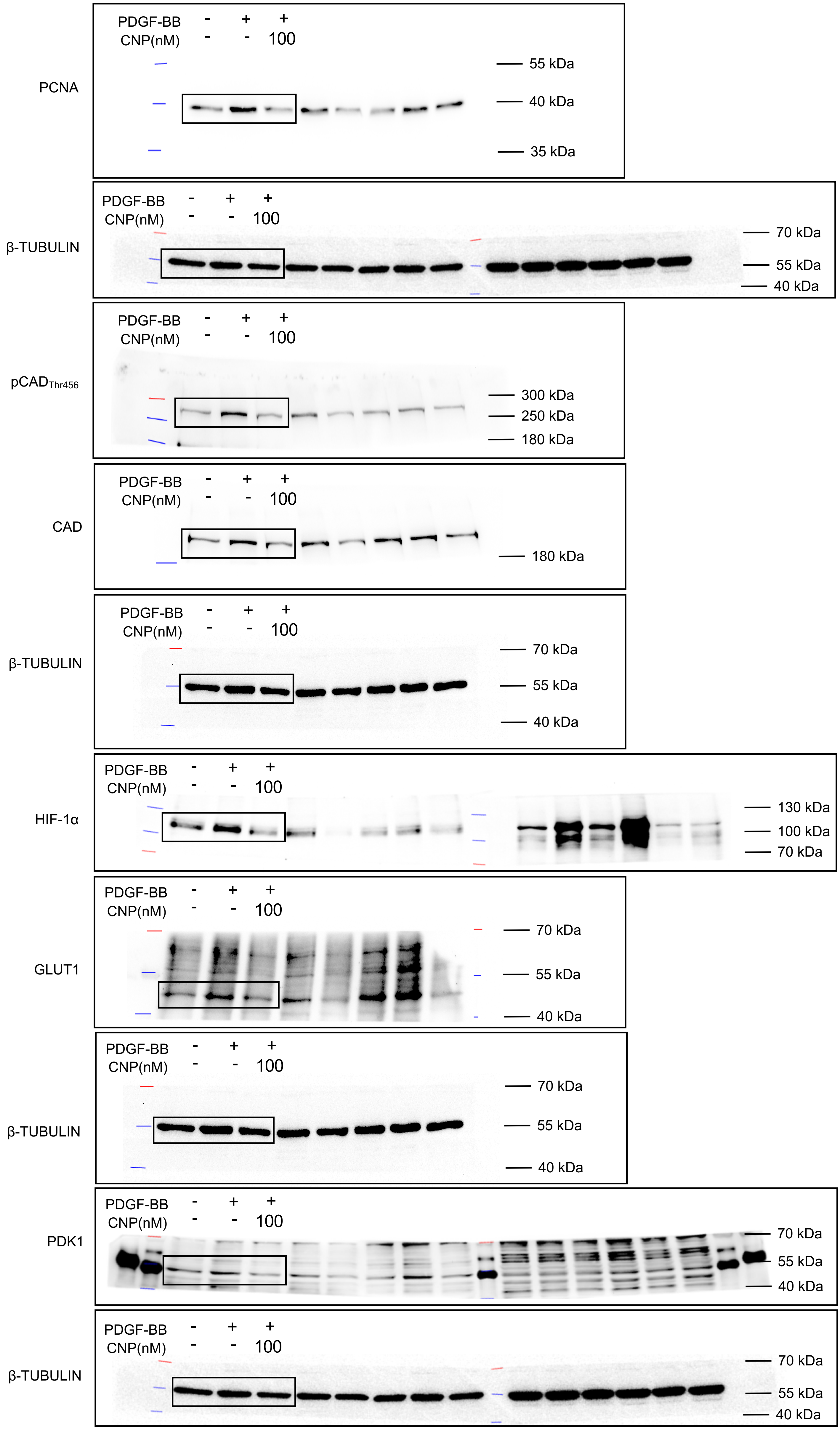

Supplementary Figure 1: Uncropped western blots

GLUT-1

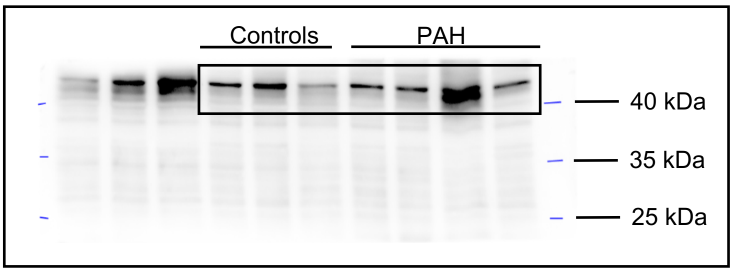

Na+-K+-
ATPase

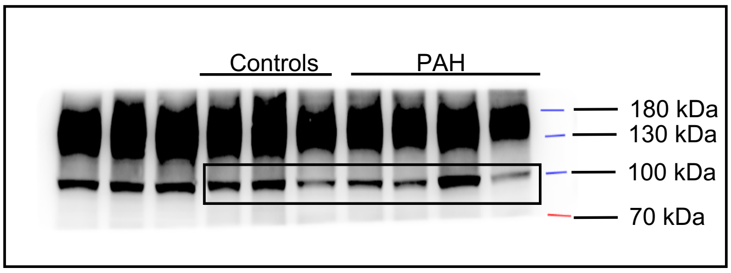

Supplementary Figure 4: Uncropped western blots

Supplementary Figure 4a

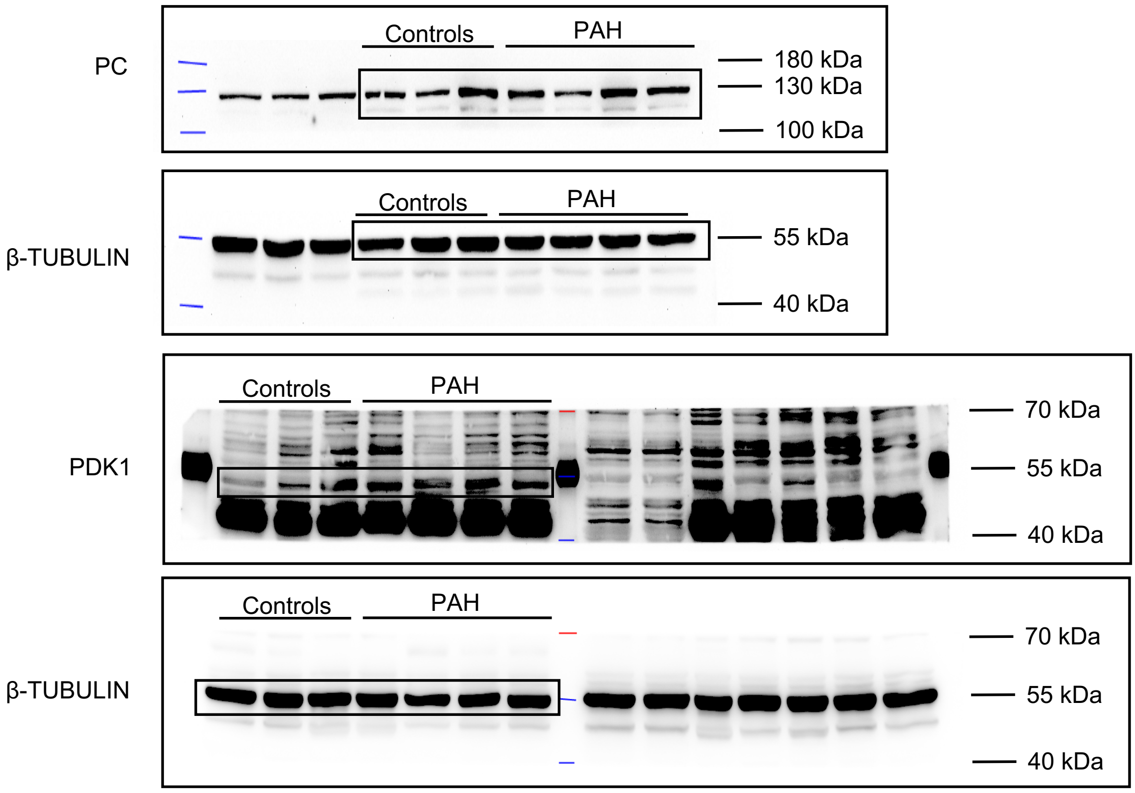

Supplementary figure 5: Uncropped western blots

Supplementary Figure 5a

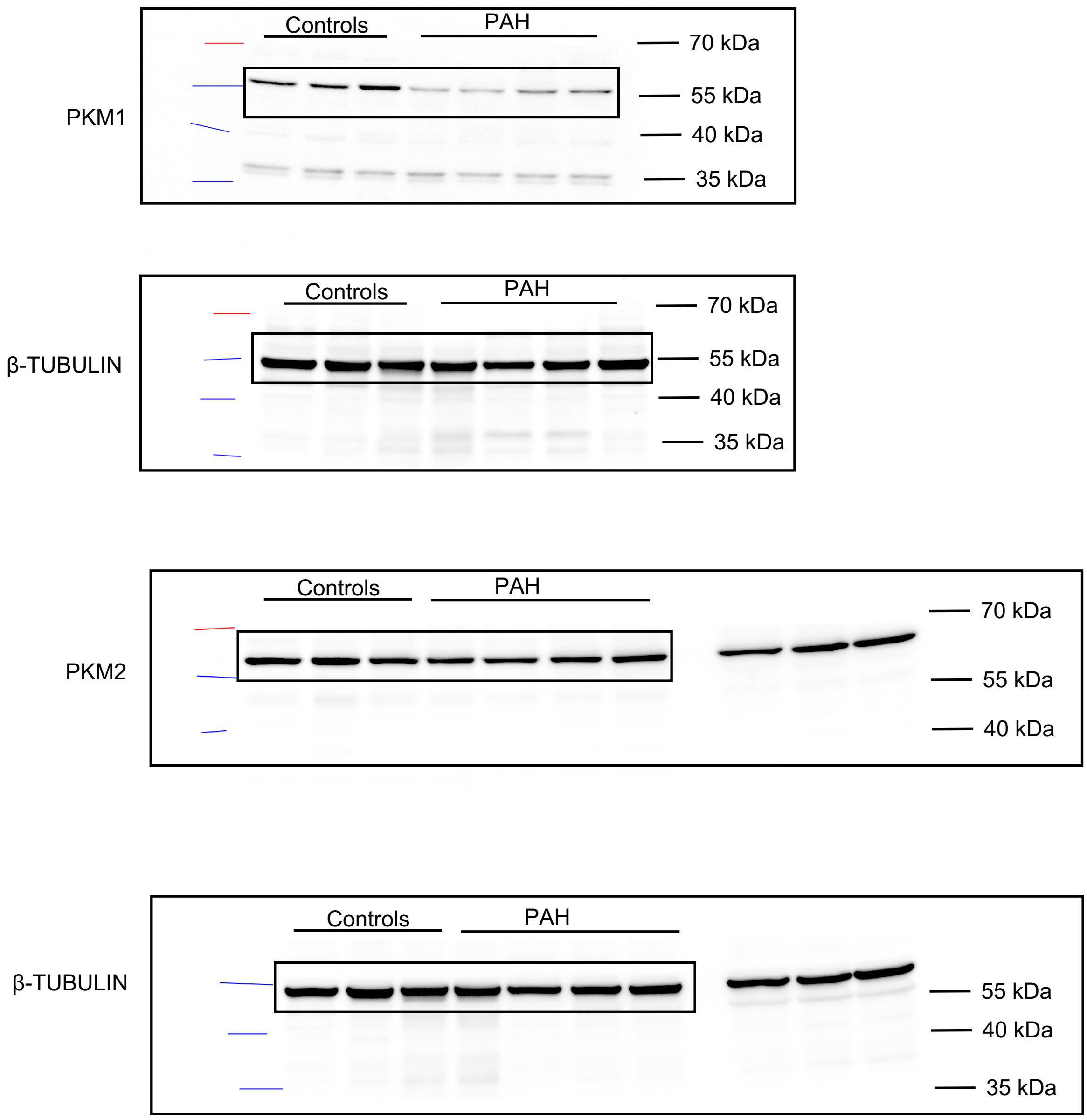

Supplementary figure 7: Uncropped western blots

Supplementary Figure 7a

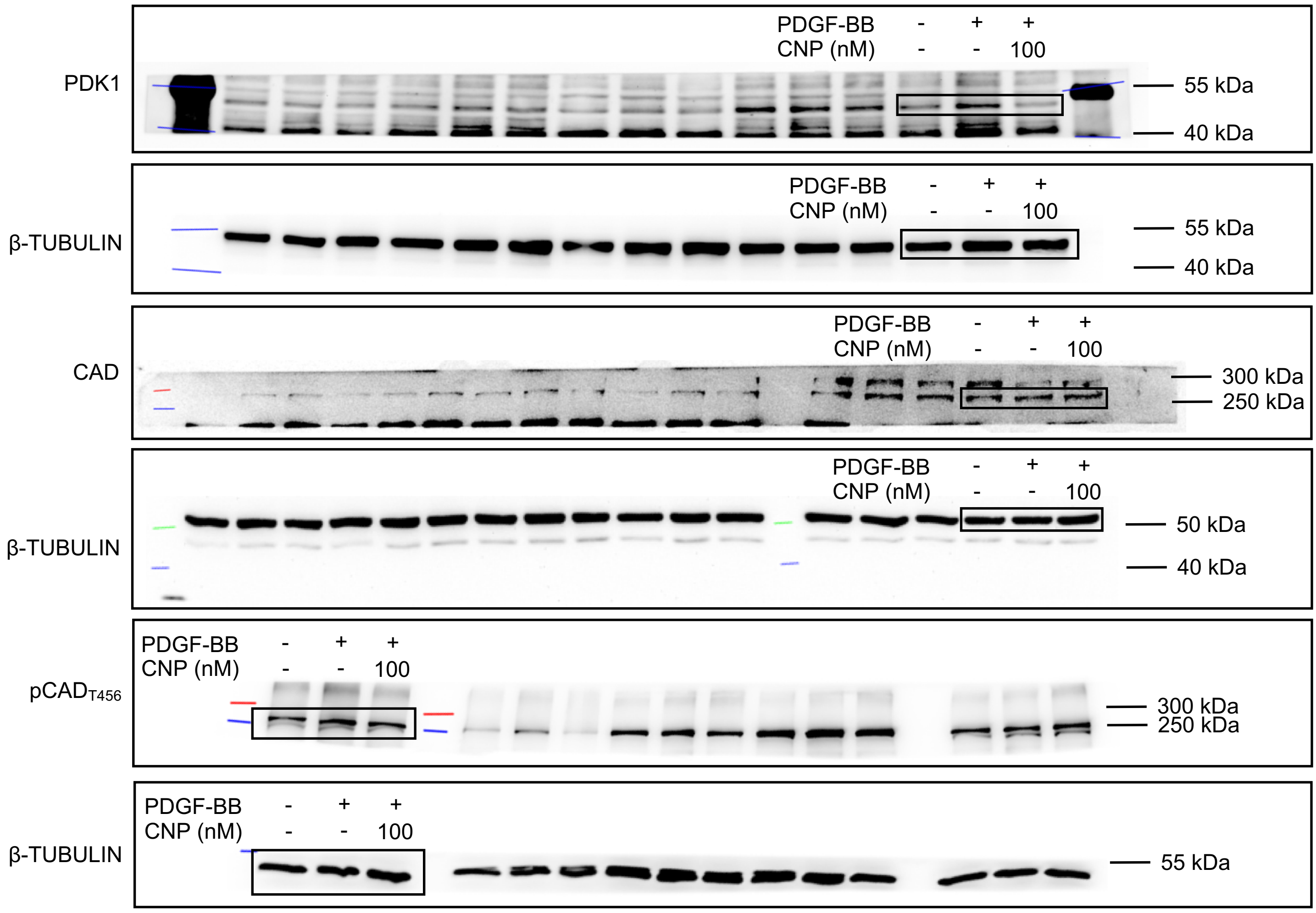

Supplementary Figure 7b

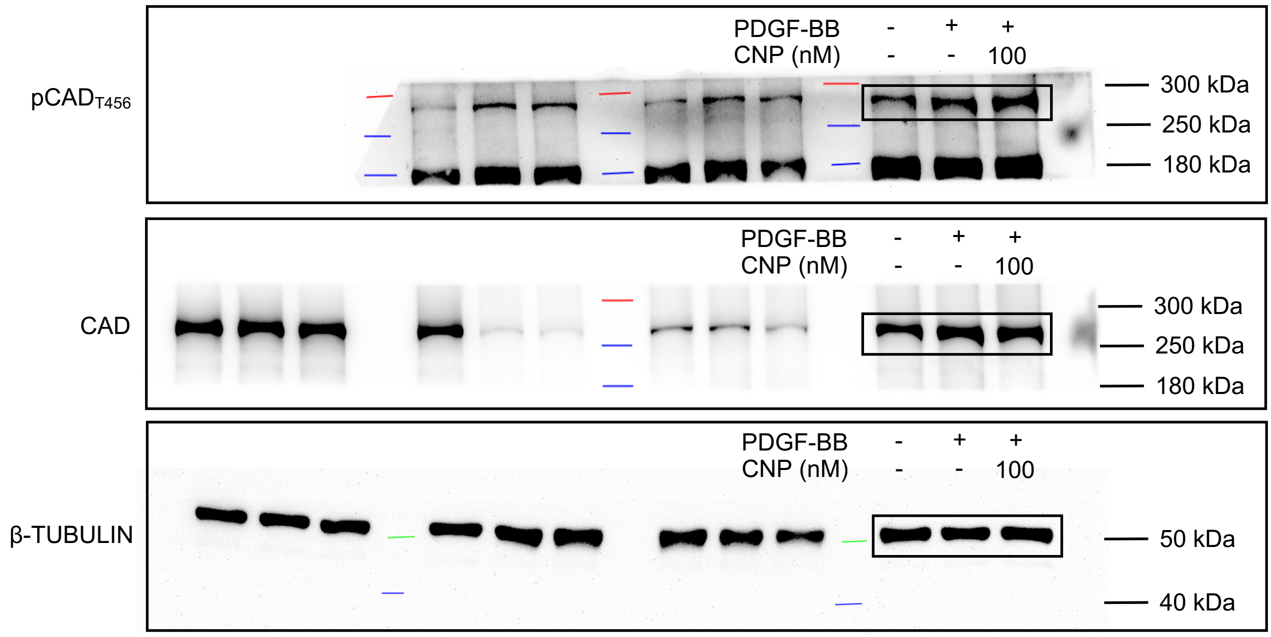

Supplementary figure 8: Uncropped western blots

Supplementary Figure 8b

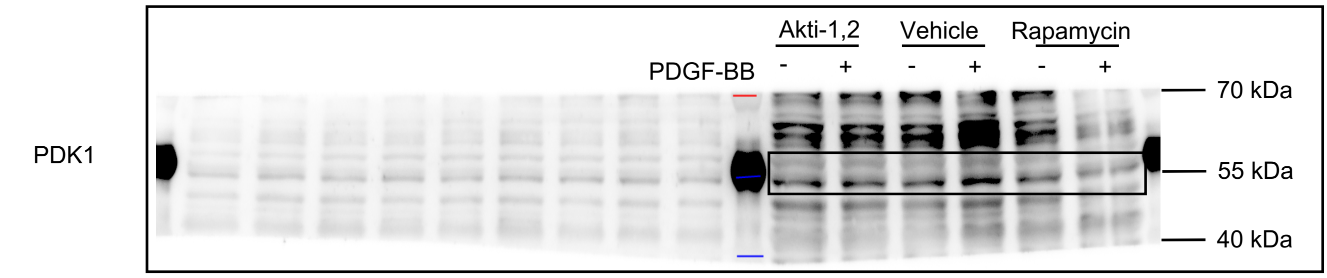

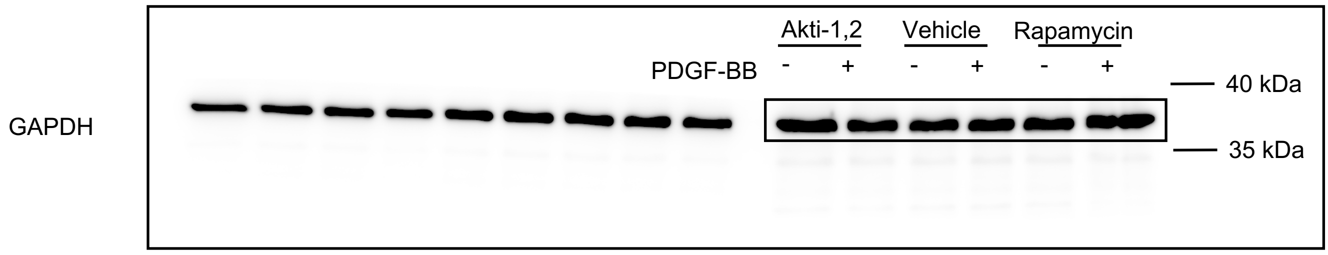

Supplementary Figure 8c

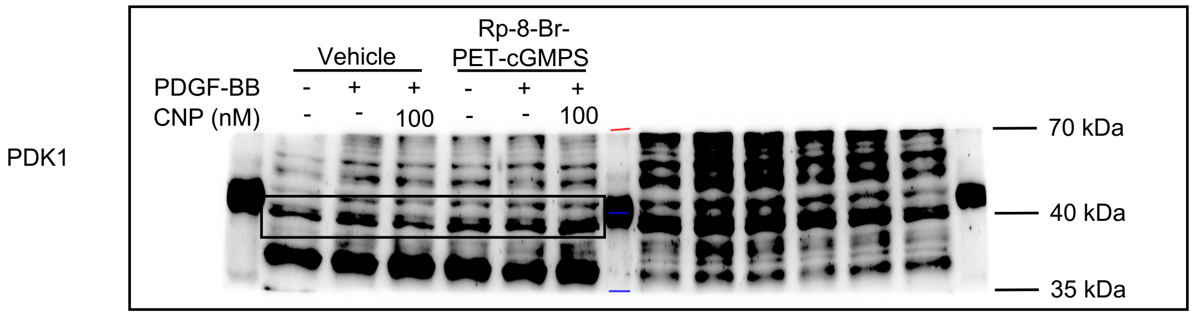

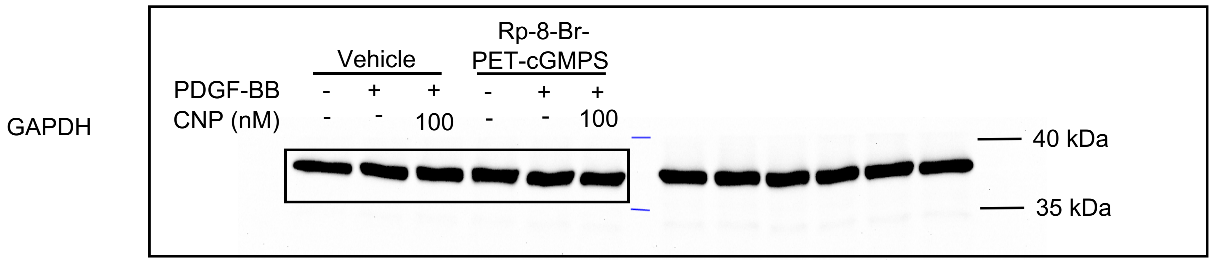

Supplement: Supplementary file 1 — Supplementary material [file 42003_2025_8661_MOESM1_ESM.pdf]
