# Supplementary material for: C-type natriuretic peptide attenuates enhanced glycolysis and de novo pyrimidine synthesis in pericytes of patients with pulmonary arterial hypertension
Source: Commun Biol. 2025 Aug 12;8:1199. doi: 10.1038/s42003-025-08661-0 (PMC12343844; doi:10.1038/s42003-025-08661-0)
Supplement: Supplementary file 3 — Description of Additional Supplementary Files [file 42003_2025_8661_MOESM3_ESM.docx]

Description of Additional Supplementary Files

**File name:** Supplementary Data 1

**Description:** Numerical values of the figures.
